# HPV Clearance and the Neglected Role of Stochasticity

**DOI:** 10.1371/journal.pcbi.1004113

**Published:** 2015-03-13

**Authors:** Marc D. Ryser, Evan R. Myers, Rick Durrett

**Affiliations:** 1 Department of Mathematics, Duke University, Durham, North Carolina, United States of America; 2 Department of Obstetrics and Gynecology, Duke University Medical School, Durham, North Carolina, United States of America; University of California San Diego, UNITED STATES

## Abstract

Clearance of anogenital and oropharyngeal HPV infections is attributed primarily to a successful adaptive immune response. To date, little attention has been paid to the potential role of stochastic cell dynamics in the time it takes to clear an HPV infection. In this study, we combine mechanistic mathematical models at the cellular level with epidemiological data at the population level to disentangle the respective roles of immune capacity and cell dynamics in the clearing mechanism. Our results suggest that chance—in form of the stochastic dynamics of basal stem cells—plays a critical role in the elimination of HPV-infected cell clones. In particular, we find that in immunocompetent adolescents with cervical HPV infections, the immune response may contribute less than 20% to virus clearance—the rest is taken care of by the stochastic proliferation dynamics in the basal layer. In HIV-negative individuals, the contribution of the immune response may be negligible.

## Author Summary

Worldwide, 5% of all cancers are associated with the sexually transmitted human papillomavirus (HPV). The most common cancer types attributed to HPV are cervical and anal cancers, but HPV-related head and neck cancers are on the rise, too. Even though the lifetime risk of infection with HPV is as high as 80%, most infections clear spontaneously within 1–2 years, and only a small fraction progress to cancer. In order to identify who is at risk for HPV-related cancer, a better understanding of the underlying biology is of great importance. While it is generally accepted that the immune system plays a key role in HPV clearance, we investigate here a mechanism which could be equally important: the stochastic division dynamics of stem cells in the infected tissues. Combining mechanistic mathematical models at the cell-level with population-level data, we disentangle the contributions from immune system and cellular dynamics in the clearance process. We find that cellular stochasticity may play an even more important role than the immune system. Our findings shed new light onto open questions in HPV immunobiology, and may influence the way we vaccinate and screen individuals at risk of HPV-related cancers.

## Introduction

Infection with the human papillomavirus (HPV) is responsible for a large fraction of anogenital and oropharyngeal cancers in both women and men. Over 90% of cervical cancers are caused by HPV infections, and up to 60% of squamous cell carcinomas of the vulva, vagina, anus and penis are associated with high-risk types of HPV [1]. More recently, it has been shown that infection with HPV also plays a critical role in the genesis of certain head and neck cancers, particularly in cancers of oropharynx and base of tongue [2]. The incidence of these cancers in men has been increasing over the past decade, suggesting the emergence of a virus-related cancer epidemic [3].

Even though the lifetime risk of HPV infections is as high as 80% [4], most individuals clear the virus within 1–2 years [5]. However, if infection with a high-risk type of HPV persists, the viral genes can interfere with the cellular control mechanisms and trigger neoplastic changes, which can eventually develop into an invasive carcinoma [6].

To date, several aspects of the HPV infection dynamics remain poorly understood [7, 8]. In particular, the mechanisms of virus clearance are controversial [8]. Clearance of HPV infection is usually attributed to an effective immune response, and the observation of longer clearance times in immunocompromised individuals further corroborates this assumption [9]. On the other hand, the fact that development of antibodies preventing future re-infection after clearing of the virus (known as seroconversion) occurs only partially [10–14] suggests that mechanisms other than an effective immune response may contribute to viral clearance.

**Fig 1:**
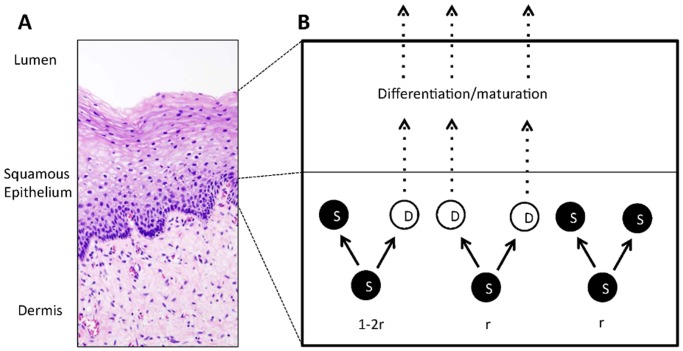
Stratified squamous epithelium. **A** H&E stain of normal cervical SSE, with squamous epithelium separating dermis from lumen (source: http://commons.wikimedia.org; work is licensed under the Creative Commons Attribution-Share Alike 3.0 Unported license). **B** In the basal layer (bottom), stem cells (solid circles) divide at rate *λ*: asymmetrically with probability 1 − 2*r*, and symmetrically with probability 2*r*. Differentiating daughter cells (empty circles) leave the basal layer at rate Γ toward the intermediate layer where they further differentiate and mature. Eventually, the top layer gets sloughed off into the lumen.

One potential contributor in the clearing of HPV that has received little attention is chance itself, or more precisely, the stochasticity of the stem cell dynamics in the infected epithelia. Across different organs (both anogenital and oropharyngeal), oncogenic types of HPV preferentially infect areas of stratified squamous epithelium (SSE), and these SSE are not just a static backdrop to the unfolding infection process [2, 15]. They have a relatively fast turnover rate and the entire thickness of the epithelium is renewed every few weeks. During the renewal process, stem cell-like progenitor cells (hereafter denoted as *S* cells) in the lowest layer of the tissue (the basal layer) produce commited daughter cells (denoted as *D* cells) that differentiate and move upwards into the intermediate and superficial layers, and eventually get sloughed off into the lumen [15], see Fig. 1. The critical role of the dynamic differentiation and maturation process in the viral life cycle is well established [16]. However, the hypothesis that stochastic dynamics in the basal layer could contribute significantly to the clearing of new infections has not been addressed elsewhere.

Until recently, the driving cellular processes in the basal layer were only poorly understood, but novel lineage tracing techniques have provided valuable insight into the stochastic dynamics of basal cells [17]. Several mouse studies have used fluorescent labeling to observe lineage dynamics over time, and have concluded that while *S* cell division is prominently asymmetric (yielding one *S* and one *D* cell), a small fraction of *S* cell divisions are symmetric, yielding either two stem cells or two differentiated daughter cells [18, 19]. Considering that HPV infections start with a small number of infected basal cells in the SSE [16], it seems plausible that these stochastic division patterns in basal cells may have an impact on the persistence properties of the infection.

To investigate the relevance of cellular proliferation patterns and tissue homeostasis on HPV infection dynamics, we develop in this study a stochastic model of HPV infection in the SSE. By explicitly accounting for the stochasticity in stem cell proliferation, as well as cytotoxic T-cell mediated elimination of infected basal cells, we investigate the potential role of chance in the viral clearing process. Combining the model with a longitudinal data set of cervical HPV infections, we provide evidence for the critical role of stochasticity in HPV clearance.

## Methods

### Model

Across affected anogenital and oropharyngeal sites, the dynamics of HPV infections are similar in nature. There is a large overlap among HPV types found in lesions of different sites, and HPV-16 is the most common type found in all HPV-related invasive cancers [20]. In addition, the viral replication strategy is essentially the same across affected sites [21, 22]. On the other hand, there are some organ-specific differences with respect to the biology of the affected stratified squamous epithelia. In fact, cervical, anal and oropharyngeal infections are usually restricted to a confined metaplastic transformation zone that separates columnar and squamous regions of the epithelium, whereas infections of e.g. the vulva, vagina and penis do not take place in such a transformation zone [23, 24]. Nevertheless, the bottom-up renewal dynamics (as explained below) of the affected epithelia are very similar, and the parametric model developed here can be applied to different tissue types by virtue of adjusting the relevant parameters, such as density of stem cells in the basal layer and regeneration time of the epithelium.

#### Homeostasis of the SSE

In physiological equilibrium, SSE (Fig. 1A) are dynamic tissues, and their entire thickness is renewed every few weeks. The renewal time Δ*T* is site-specific and varies between 3–6 weeks in the cervix [22, 25] and 2–3 weeks in the oral mucosa [26]. The regeneration is a bottom-up process as depicted in Fig. 1B, and the following dynamic model has been proposed based on lineage-tracing experiments [18, 19] (Fig. 1B). Stem cell-like progenitor cells (*S*) in the basal layer divide at a rate *λ*, and during each division, there are three possible outcomes: with probability 1 − 2*r* (where *r* ≪ 1), *S* cells divide asymmetrically into one *S* and one differentiated daughter cell (*D*); with probability 2*r*, the division is symmetric, resulting in either two *S* or two *D* cells. Since space in the basal layer is limited, *D* cells leave the basal layer at rate Γ, and move upwards into the intermediate layers. Once fully matured and differentiated, *D* cells have reached the superficial layers, where they are eventually shed off to make space for new cells. Mathematically, these dynamics are summarized as a two-type branching process, see also Fig. 1B,

$$\begin{array}{c} S\overset{\lambda }{\to }\left\{\begin{array}{c} S+S\;\;\text{with}\;\text{probability}\;r\\ S+D\;\;\text{with}\;\text{probability}\;1-2r\\ D+D\;\;\text{with}\;\text{probability}\;r \end{array}\right.\;D\overset{\Gamma }{\to }\emptyset , \end{array}$$ (1)

where ∅ signifies cell death [18, 27]. In homeostasis, the basal layer consists of a conserved fraction *ρ* of S cells, which means that proliferation and migration rates satisfy the relationship *ρλ* = Γ(1 − *ρ*). Since the *S* cells in (1) undergo a critical branching process, their progeny will eventually go extinct (see also the discussion of S* cells below). However, since the basal layer is not compartmentalized and there is a large pool of *S* cells, this is very unlikely to occur within a human life time. In addition, it has been shown that there are regulatory mechanisms for stem cell fate [19, 28], and it is conceivable that similar mechanisms prevent the total number of *S* cells to fluctuate significantly. Finally, even though this model was originally developed based on mouse experiments, it has since been corroborated in human SSE [29].

#### HPV infection dynamics

New HPV infections arise in the basal layer of the SSE: physical ruptures in the tissue allow virions to reach the bottom layer of the SSE, where they infect residing *S* and *D* cells [16]. Due to the high turnover rate of SSE, non-dividing and upward moving *D* cells are lost from the epithelium within a few weeks, and hence persistent infections require the infection of *S* cells. Hereafter, we denote infected *S* and *D* cells by S* and D*, respectively. Furthermore, we denote by *n*_*X*_(*t*) the number of cells of type *X* ∈ {*S*, *D*, S*, D*} present at time *t*. Since the viral count is kept at very low copy numbers (10–100) in the basal layer, and there is only minimal viral gene expression [30], we assume that the host cell dynamics are not affected by the presence of the virus. In particular, it has been shown that HPV-infected cells only acquire a selective growth advantage once the viral DNA has been integrated into the host DNA [31], which occurs at later, symptomatic stages of the infection. Consequently, the dynamics of infected cells in the early stages are still governed by (1), with *S* and *D* replaced by S* and D*, respectively. From this it is easy to see that the dynamics of the S* cell population in the basal layer are governed by the continuous-time critical branching process

$$\begin{array}{c} S^{*}\overset{2r\lambda }{\to }\left\{\begin{array}{cc} S^{*}+S^{*} & \;\;\text{with}\;\text{probability}\;1/2,\\ \emptyset & \;\;\text{with}\;\text{probability}\;1/2. \end{array}\right. \end{array}$$ (2)

It follows from the theory of branching processes [32] that the probability an infected clone (starting from one infected cell) survives until time *t* is

$$\begin{array}{c} \mathbb{P}\left(n_{S^{*}}\left(t\right)>0\right)=\frac{1}{1+r\lambda t}. \end{array}$$ (3)

In particular, the clone will die out with probability 1, though the time until this occurs has infinite expected value.

#### Immune response

Even though HPV is equipped with molecular mechanisms that facilitate immune evasion after infection, it is generally assumed that clearance of the virus is the result of a successful immune response [25, 33]. Initially, detection of the infection triggers an innate immune response which targets the virions that are released at the surface, as well as infected cells in the superficial layers. However, neither of these responses can permanently clear the virus: elimination of basal cells require the presence of HPV-specific cytotoxic T-cells which are recruited during an adaptive immune response [24]. To model the specific targeting of infected basal cells, we need to introduce T cell mediated elimination of infected S* and D* cells. For this purpose, we assume that the number of T cells recruited is proportional to the number of infected cells in the basal layer, *n*_S*_ + *n*_D*_. This implies that each infected cell is targeted at a constant rate *μ*, referred to hereafter as the immune capacity,

$$\begin{array}{c} S^{*},\;D^{*}\overset{\mu }{\to }\emptyset . \end{array}$$ (4)

**Fig 2:**
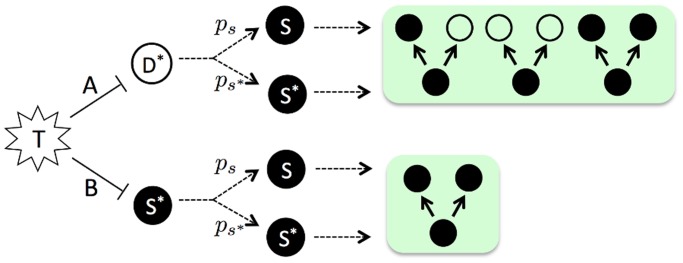
Replacement dynamics. **(A)** T-cell elimination of an infected daughter cell (D*) triggers division of an *S* or S* cell with probabilities *p*_*s*_ and *p*_s*_, respectively. The division event is identical to spontaneous division in Fig. 1B. **(B)** T-cell elimination of an infected stem cell S* triggers division of an *S* or S* cell. However, as required by homeostatic equilibrium of the stem cell compartment (on average), the division is assumed to yield two identical stem cells, see text for details.

Of note, *μ* represents the effective immune capacity, accounting both for the strength of the mounted immune response, and the likelihood of immune cells to detect and neutralize infected basal cells. Finally, since the number of basal cells is assumed to be conserved on average, the eliminated cell needs to be replaced. Therefore, elimination of an infected cell triggers proliferation of an *S* cell with probability *p*_*S*_, or of a S* cell with probability *p*_S*_ = 1 − *p*_*S*_, see Fig. 2. The nature of these probabilities will be discussed in *Results*.

#### Replacement dynamics

Next, we describe the replacement dynamics that ensue after elimination of infected basal cells. The underlying premise for the following replacement rules is conservation (on average) of basal stem cells. While there is, to our knowledge, no direct experimental evidence for conservation of basal stem cells during viral clearance, it has been shown that stem cell fate can change temporarily and reversibly after perturbation [19], see also [28]. With this in mind, and as illustrated in Fig. 2A, we assume that D* cell elimination triggers division of a stem cell (infected or uninfected) in asymmetric or symmetric fashion, identically to the spontaneous division events depicted in Fig. 1B. On the other hand, S* cell elimination is assumed to trigger a symmetric division, resulting in two identical *S* copies if an *S* cell is triggered, and into two S* copies if an S* cell is prompted to divide (Fig. 2B). In fact, if S* division were to trigger asymmetric division of either an *S* or an S* cell, this would violate our assumption of epithelial cell homeostasis because the total number of stem cells in the tissue would not be conserved over time.

#### Complete model

In summary, the infection dynamics in the basal layer are described by a 4-dimensional continuous-time Markov process with variables *S*, S*, *D* and D*. The process is governed by the spontaneous division and migration events as in Fig. 1B, in conjunction with the immune-mediated events depicted in Fig. 2. Furthermore, we shall assume that each infection starts with an inoculum of *n*_0_ infected stem cells S* at time *t* = 0.

### Data

#### Ethics statement

The longitudinal REACH data set [34] used in this study is publicly available and distributed by Sociometrics Corporation (http://www.socio.com/reachdata.php). Access to the non-sensitive part of the data (standard data) used in our study was granted by Sciometrics Corporation without IRB or other ethics approval.

#### Data description and exclusion criteria

To calibrate the models we used longitudinal infection data from *The Reaching for Excellence in Adolescent Care and Health (REACH)* project of the Adolescent Medicine HIV/AIDS Research Network [34]. Between 1996–2000, the REACH study followed 578 HIV-infected and HIV-uninfected adolescents (ages 13–18) in 13 US cites. A detailed description of the study is found in [35], see also [36]. In particular, the 411 female participants in the cohort were tested for HPV every 6 months, and we extracted the corresponding longitudinal data set from the master file. Not all data points were suitable for our purposes, so we made exclusions according to the following criteria: 31 participants did not test positive for HPV during the study; 31 participants had less than 2 valid HPV tests; 15 participants had less than one valid HPV test after the first HPV positive test; 6 participants had missing visit dates; 15 participants had incomplete information about the HPV subtypes. Among the remaining 313 females included in our analysis, 212 were HIV-positive, and 121 were HIV-negative. None of the participants changed HIV-status during the study period.

## Results

### Estimates from the parametric data analysis

The first objective was to combine the model introduced in *Methods* with the REACH data set to obtain estimates of the proliferation dynamics, the immune capacity, and the number of initially infected basal cells. For this purpose, we made the assumption of a well-mixed basal layer: upon removal of an infected cell, division of an *S* cell occurs with probability *p*_*S*_ = *n*_*S*_/(*n*_*S*_ + *n*_S*_), and division of an S* cell with probability *p*_S*_ = *n*_S*_/(*n*_*S*_ + *n*_S*_). In other words, we assumed that the spatial arrangement of cells in the 2D basal layer can be ignored (the opposite end of the spectrum—a spatially clustered population of infected cells—is discussed below). Since the relative size of the infected population compared to the entire basal layer is small throughout the infection, *n*_*S*_ ≫ *n*_S*_, we can approximate *p*_*S*_ ≈ 1 and *p*_S*_ ≈ 0. As outlined in section 2 in S1 Text, it follows that the S* cell dynamics reduce to a subcritical branching process,

$$\begin{array}{c} S^{*}\overset{}{\to }\left\{\begin{array}{cc} S^{*}+S^{*} & \;\;\text{at}\;\text{rate}\;\lambda r,\\ \emptyset & \;\;\text{at}\;\text{rate}\;\lambda r+\mu . \end{array}\right. \end{array}$$ (5)

The probability of survival to time *t* for this process is, according to results in [32],

$$\begin{array}{c} \mathbb{P}\left(n_{S^{*}}\left(t\right)>0\right)=\frac{1}{1+\frac{\lambda r+\mu }{\mu }\left(e^{\mu t}-1\right)}. \end{array}$$ (6)

In particular, addition of the immune capacity transforms the ∼ 1/*t* decay in (3) into an exponential decay.

Next, we used the longitudinal HPV data from the REACH study to infer the model parameters via maximum likelihood estimation (MLE). Thereby, we faced the issue of non-identifiability of the model, a common problem in statistical inference. To understand where these issues arise, we first consider the probability density function *f* for the persistence time of the infection (see section 3 in S1 Text for its derivation)

$$\begin{array}{c} f\left(t\right)=\left\{\begin{array}{cc} \frac{n_{0}{\left(\lambda r\right)}^{n_{0}}t^{n_{0}-1}}{{\left(1+\lambda rt\right)}^{n_{0}+1}}, & \mu =0,\\ \frac{n_{0}A^{n_{0}}\mu e^{\mu t}{\left(e^{\mu t}-1\right)}^{n_{0}-1}}{{\left(1+A\left(e^{\mu t}-1\right)\right)}^{n_{0}+1}}, & \mu >0, \end{array}\right. \end{array}$$ (7)

where *A* ≡ (*λr* + *μ*)/*μ*, and *n*_0_ is the initial number of infected stem cells. From (7) we see that the values of *λ* and *r* cannot by inferred individually, and the best we can do is infer their product, *α* ≡ *λr*. Even though there are no further apparent identifiability issues, we found that for *n*_0_ large enough, the density (7) only depends on the ratio *α*/*n*_0_ (see section 4 in S1 Text). As a consequence, *α* and *n*_0_ cannot be inferred individually, and we perform the inference over *μ* and *n*_0_ for fixed values of *α*, across a prior range of biologically meaningful values *α* ∈ [0.01, 0.25] *d* ^−1^(see section 5 in S1 Text for a justification of this range).

In addition to the identifiability issues, the MLE required the derivation of a non-standard likelihood function that takes into account the different combinations of data types: infections were either present at the time of the first visit (prevalent infections), or they were initiated after the first visit (incident infections); some individuals were lost to follow-up before clearing the virus (right-censoring), and both the time of initiation and the time of clearance were only determined up to the between-visit intervals (interval-censoring). The derivation of the corresponding likelihood function is found in section 3 in S1 Text.

A final comment regarding parameter inference concerns the interpretation of negative test results. In fact, it has been shown that longitudinal HPV studies bear a significant amount of misclassifications due to short-term variation [37], and that apparently cleared infections can reappear after variable amounts of time [38, 39]. The time before reappearance of seemingly cleared infections could be interpreted as a latency period during which the infection temporarily regresses to subdetection levels. However, molecular evidence for this latency mechanism has so far only been established in animal models [40]. Therefore, we decided to interpret the first of two consecutive negative test results as the time of clearance of the infection.

**Fig 3:**
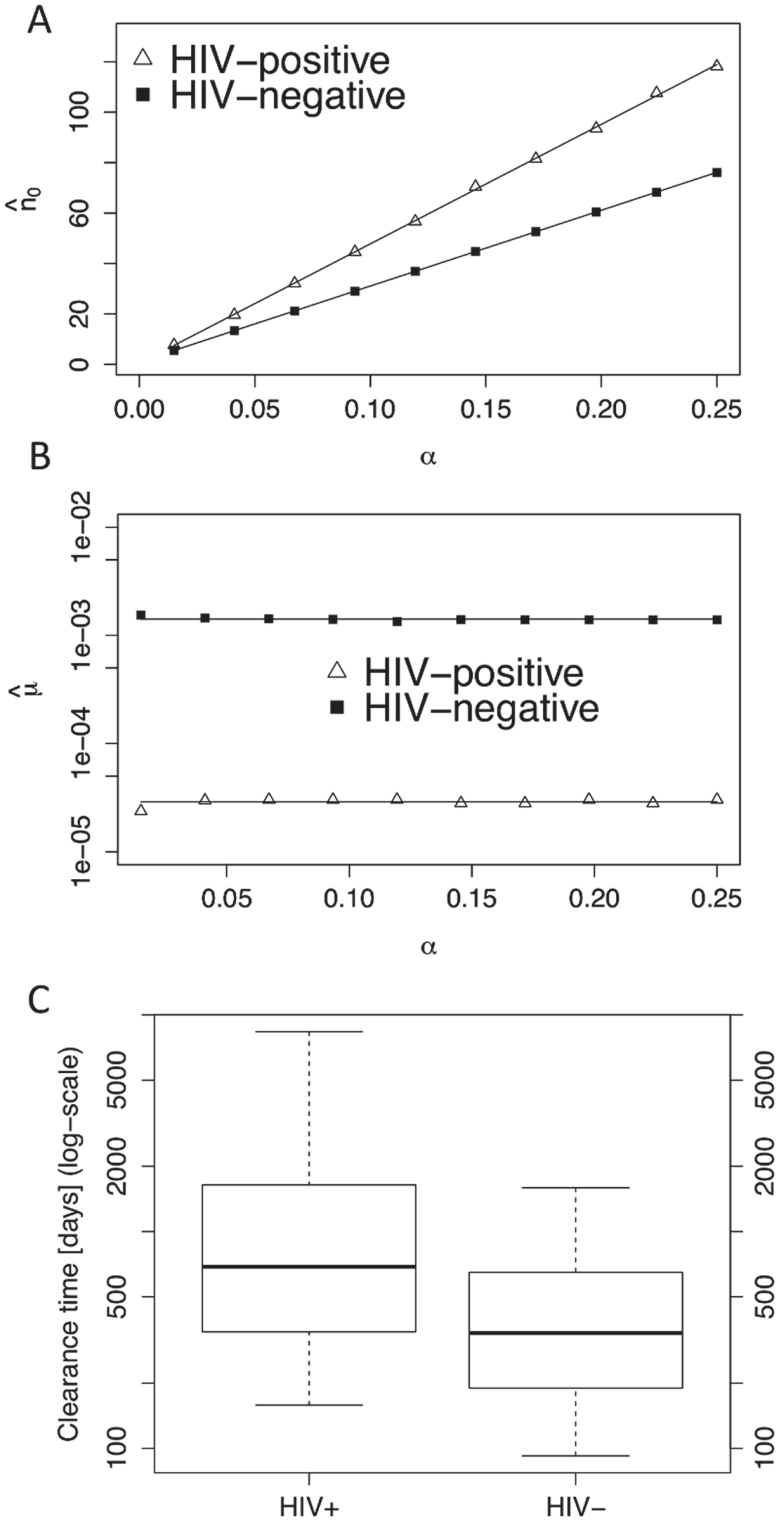
Parameter inference and clearance time distributions. **A, B** MLE inference for the initial number of infected cells *n*_0_ and immune capacity *μ* for the HIV-negative and HIV-positive cohorts, over the prior range of *α*. Estimators are denoted by a (^) symbol. Due to identifiability issues (see text), the estimators ${\hat{n}}_{0}$ are linear functions of *α*, whereas the estimators $\hat{\mu }$ are constant over the prior range of *α*. The ${\hat{n}}_{0}$ estimates are similar between the HIV-negative and HIV-positive cohorts (A), but there is a 100-fold difference for the $\hat{\mu }$ estimates (B). **C** The parametric clearance time distributions for both cohorts are derived using equation (7) and the estimates from panels A and B. The distributions are insensitive to the choice of *α*, see section 6 in Text SI (plots shown for *α* = 0.14). The band inside the box is the median, the bottom and top of the box are 1st and 3rd quartile, respectively, and the whiskers correspond to the 5th and 95th percentiles, respectively.

The inference results are summarized in Fig. 3A-B. In what follows, the maximum likelihood estimates are denoted by a hat (^) on the parameter name, and subscripts (−) and (+) are used to refer to the HIV-negative and HIV-positive cohorts, respectively. As explained above, the number of initially infected cells *n*_0_ is a linear function of *α*, which varies over the prior range [0.01, 0.25]. The inferred ranges for the initial number of infected cells are ${\hat{n}}_{0,-}(\alpha )\in$ [5, 80] in the HIV-negative cohort, and ${\hat{n}}_{0,+}(\alpha )\in$ [5, 120] in the HIV-positive cohort, see Fig. 3A. Across the prior range of *α*, the inferred number of initially infected cells is slightly higher (but of the same order of magnitude) in HIV-positive compared to HIV-negative individuals: ${\hat{n}}_{0,+}(\alpha )>{\hat{n}}_{0,-}(\alpha )$, for all *α*. To our knowledge, there is no experimental data that would allow us to assess the validity of these model predictions. Regarding the immune capacity *μ*, we find a stark difference between the cohorts: the estimated capacity ${\hat{\mu }}_{-}$ in the HIV-negative cohort $\left({\hat{\mu }}_{-}=1.4\cdot 10^{-3}d^{-1}\right)$ is two orders of magnitude larger than the estimated capacity ${\hat{\mu }}_{+}$ in the HIV-positive cohort $\left({\hat{\mu }}_{+}=3\cdot 10^{-3}d^{-1}\right)$, see Fig. 3B. In particular, the estimates ${\hat{\mu }}_{+}$ and ${\hat{\mu }}_{-}$ are constant over the prior range of *α*.

Using the inferred parameter values ${\hat{\mu }}_{+}$ and ${\hat{\mu }}_{-}$ for the immune capacity, and the inferred ranges ${\hat{n}}_{0,-}(\alpha )\in$ and ${\hat{n}}_{0,+}(\alpha )\in$ for the number of initial cells, we then derived the parametric clearance time distributions according to (7), see Fig. 3C. Since the clearance time distributions were found to be insensitive to *α* over the prior range (see section 6 in Text SI), the distribution in Fig. 3C is only shown for an intermediate value of *α* = 0.14. Due to the reduced immune capacity in the HIV-positive cohort, its median clearance time is considerably larger (689 days) than the median time in the HIV-negative cohort (340 days).

### Stochasticity vs immune response

**Fig 4:**
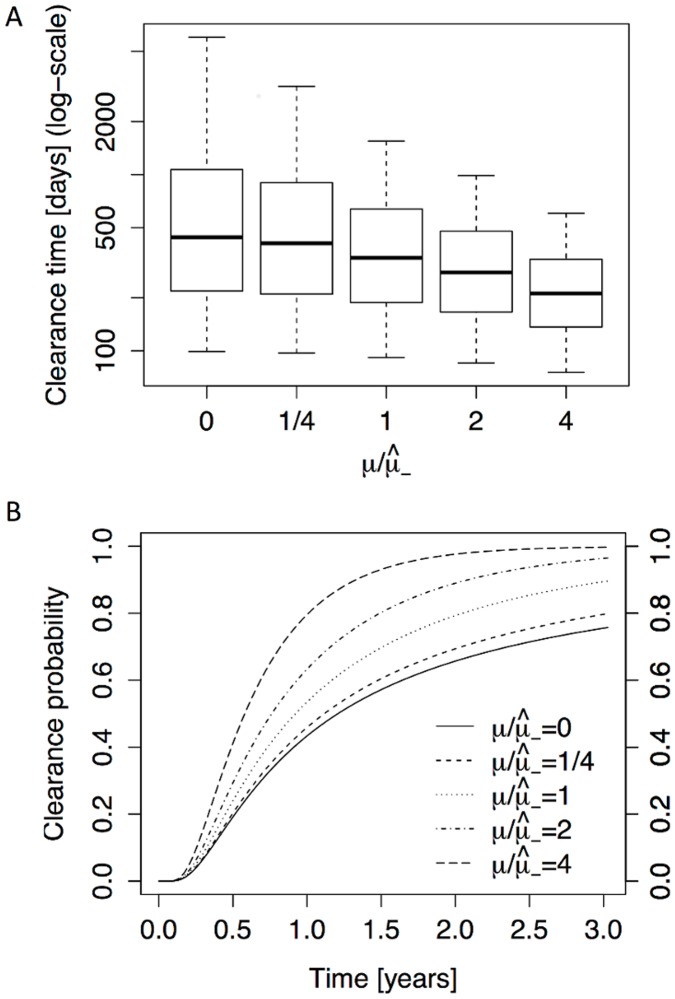
Immune capacity in the branching process model. **A** The model-derived distribution of the clearance time is shown as a function of varying immune capacity *μ* (note that ${\hat{\mu }}_{-}$ corresponds to the immune capacity of HIV-negative individuals). The box-and-whisker plots correspond to the 5th, 25th, 50th, 75th and 95th percentiles, see caption of Fig. 3 for details. **B** The clearance probability as a function of time is shown for different levels of immune capacity.

The main goal of this study was to assess the relative roles of stochastic cell dynamics and immune response in the process of HPV clearance. Therefore, we compared the model-based persistence distributions for varying immune capacities *μ*. As shown in Fig. 4A, the median time to clearance is a decreasing function of *μ*, and the distributions become more localized with increasing *μ*. However, comparing the distributions for *μ* = 0 and $\mu /{\hat{\mu }}_{-}=1$ (where $\mu ={\hat{\mu }}_{-}$ is the estimated immune capacity of HIV-negative individuals), the contribution of the immune response appears to be small in comparison to the contribution of the stochastic cell dynamics (compare the box plots for *μ* = 0 and $\mu /{\hat{\mu }}_{-}=1$ in Fig. 4A). This is particularly clear when plotting the clearance probability as a function of time as shown in Fig. 4B. In particular, comparing the ($\mu /{\hat{\mu }}_{-}=0$)-curve with the ($\mu /{\hat{\mu }}_{-}=1$)-curve after 2 years, the clearance probability without immune response (0.66) is only ∼ 17% smaller than the clearance probability with normal immune capacity (0.79). In other words, the stochastic dynamics contribute to as much as ∼ 83% of the viral clearing mechanism in healthy individuals, and the contribution from the immune system is comparatively small.

### Space and the impact of clustering

The subcritical branching process model above was derived under the assumption of a well-mixed basal layer where infected cells are surrounded primarily by susceptible cells. In this situation, elimination of an infected cell prompts division of an *S* cell with high probability, justifying the approximation *p*_*S*_ = 1 − *p*_S*_ = 1. As a consequence, the persistence distribution could be derived analytically (6), rendering the model amenable to MLE inference. To assess whether the ensuing results were an artifact of the well-mixing assumption, we developed the following alternative model that takes into account the spatial clustering of infected cells.

If we assume that the initial cell population is subject to tight clustering, then radial symmetry implies growth in the form of a radially expanding disk in the basal layer. That is, all the infected cells are inside the disk, whereas the outside is populated only by uninfected cells. Since the number of D* cells is roughly proportional to the number of S* cells (see section 2.2 in Text SI), the disk radius is proportional to $\sqrt{n_{S^{*}}}$. Accordingly, whenever an infected cell in the interior of the disk is eliminated by a T-cell, the probability to trigger an *S* cell division is given by the ratio of disk circumference to disk area: $p_{S}=\min\;\left\{1/\sqrt{n_{S^{*}}},1\right\}$ and *p*_S*_ = 1 − *p*_*S*_. Under these assumptions, the S* cell dynamics are now decoupled from the *S* cell dynamics, but they still depend on the D* cell dynamics, see also section 2 in S1 Text for details.

**Fig 5:**
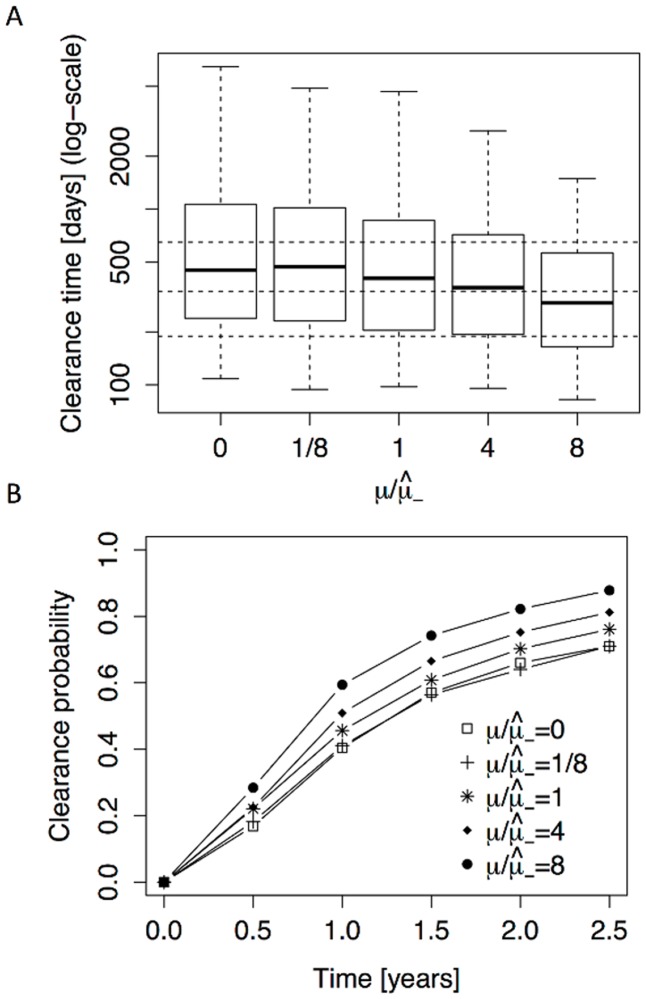
Immune capacity in the spatial model. **A** The model-derived clearance time distribution is shown as a function of varying immune capacity *μ* for the spatial model. The box-and-whisker plots correspond to the 5th, 25th, 50th, 75th and 95th percentiles, see capture of Fig. 3. The dotted lines are the three quartiles from $\mu ={\hat{\mu }}_{-}$ in Fig. 4A. **B** The clearance probability as a function of time is shown for different levels of immune capacity. All results in this figure are estimates based on 1000 simulations per *μ*-value.

Since closed-form expressions for the clearance time distributions are out of reach for this model, even with the approximation, we resorted to simulations. As in Fig. 4A for the well-mixed model, we investigated the impact of increasing immune capacity *μ* on the clearance time distribution in Fig. 5A. We make the following observations. First, time to clearance is generally longer in the branching process model: the three dotted horizontal lines correspond to the three quartiles for the $\left(\mu /{\hat{\mu }}_{-}=1\right)$-distribution in Fig. 4. Only for the $\left(\mu /{\hat{\mu }}_{-}=8\right)$-distribution, which corresponds to an 8-fold increase in immune capacity, are all three quartiles of the spatial model below the corresponding quartiles of the branching process model. Second, the impact of the immune capacity on the clearance time for the clustered model is even weaker than in the well-mixed model. Whereas the well-mixed model yields a decrease in median time to clearance for increasing *μ*, small *μ* values yield a slight increase in median clearance time for the spatial version. This is due to the fact that, in contrast to the branching process model, elimination of an infected cell can trigger division of an S* cell (with probability *p*_S*_ > 0), therefore compensating for the loss of the infected cell and delaying clearance. The relative insensitivity of the persistence time distribution to *μ* is further illustrated in Fig. 5B, where we observe that the clearance probability is only slightly increased for small *μ* values. Finally, since the prior estimates of several model parameters have a relatively large interval of uncertainty (see section 5 in S1 Text), we performed a combined sensitivity analysis. By means of a Monte-Carlo simulation (with the parameters *r*, *α*, *ρ* and *μ* drawn from their prior ranges), we computed the corresponding persistence time distribution, and found that it did not substantially differ from the fixed parameter distribution (see section 7 in S1 Text for details).

## Discussion

Clearance of anogenital and oropharyngeal HPV infections has primarily been attributed to a successful adaptive immune response. To date, little attention has been paid to the potential role of homeostatic cell dynamics in clearing HPV infections. In this study, we combined mechanistic mathematical models at the cellular level with epidemiological data at the population level to disentangle the respective roles of immune capacity and cell dynamics in the clearing mechanism. Our results suggest that chance—in form of the stochastic dynamics of basal stem cells—plays a critical role in the elimination of HPV-infected cell clones. In particular, we found that in individuals with normal immune capacity (HIV-negative cohort), the immune response may contribute to less than 20% of the clearing task overall—the rest is taken care of by the random succession of symmetric and asymmetric stem cell divisions. Furthermore, in immunocompromised individuals (HIV-positive cohort) the contribution of the immune response is likely to be negligible.

Based on our results, we may be able to shed new light onto questions currently debated in the literature. First, in view of the high prevalence of HPV infections and the relatively small risk of persistent infections that eventually lead to malignant disease, the identification of predictive markers for persistence would be valuable [8]. However, if stochasticity does indeed play a key role in viral clearance, and if the major difference between individuals who clear effectively and individuals who develop persistent infections is largely a matter of chance, then there may not be any predictive markers to discover. Hence, we may want to rephrase the question, and ask if there is a way of modulating the cellular dynamics to achieve an increase in the clearance probability. Our results suggests that by increasing either the probability of a symmetric division (*r*) or the proliferation frequency (*λ*) through a locally administered drug, time to clearance and risk of progression could be substantially reduced.

Second, the suggested clearing mechanism could provide an alternative explanation for the correlation between long-term use of combined oral contraceptives and increased risk of persistent infections and cervical cancer [41]. Since estrogen stimulates [42] and progesterone inhibits [43] epithelial proliferation, it seems plausible that a decrease in cervical proliferation could be caused directly via increased progesterone levels, and indirectly via loss of the estrogenic mid-cycle peak. The resulting decrease in proliferation (smaller *λ*) would imply an increase in time to clearance and a higher risk of progression to cancer. While the same reasoning would imply an increased risk of cervical cancer in progestin-only users, the effect of progestin-only contraceptives on HPV persistence and cervical cancer development is less consistent in the literature [44, 45]. This highlights the need for future research into the influence of sex steroids on the natural history of oncogenic HPV infection.

Finally, the suggested model of chance-driven clearance is interesting in view of the ongoing debate about viral latency [46–48]. To date, the existence of latent infections has been demonstrated in animal models, and it is assumed to occur in HPV infections as well. The current theory of latency is based on the assumption that the virus stays present inside long-lived basal stem cells [48]. But while the notion of such long-lived, asymmetrically dividing and slow-cycling stem cells is consistent with a theory of epithelial homeostasis developed in the 1970’s [49], it is not aligned with the new paradigm that is based on fast-cycling stem cells that divide both asymmetrically and symmetrically [18, 19, 29]. According to our model, which is based on this more recent theory of homeostasis, viral latency is again a stochastic phenomenon and occurs if the number of infected cells becomes very small (latent period) before growing back to a detectable size. A more thorough discussion of the latency issue will be the subject of future work.

While population-level models of HPV transmission and progression are commonly used by epidemiologists and health economists, only few groups have developed mathematical models of HPV infection at the tissue level. In two recent studies [50, 51], deterministic (partial) differential equation models were used to study evolutionary and ecological aspects of HPV infections and competition between coexisting HPV types. To our knowledge, we are the first to develop a stochastic model of HPV infection that couples stem cell dynamics with viral infection and immune response. In addition, the methods introduced here provide a useful tool in the parametric analysis of longitudinal data sets that contain both prevalent (present at study begin) and incident (initiation after study begin) infections, as well as right-censoring (study exit before viral clearance) and interval-censoring (duration of infection only known up to an interval). In fact, 70% of the individuals in the analyzed REACH data set had an unknown time of initiation, rendering conventional nonparametric approaches problematic (see section 1 in S1 Text for details). Thanks to the mechanistic models introduced and analyzed in this study, we were able to account for the unknown time lapse between infection initiation and study entry. Finally, the approach employed in this study may prove useful in other situations. In fact, mathematical models at the tissue-level are often difficult to parametrize because sample sizes in pathology studies are generally small and exhibit large between-patient variation. By combining longitudinal population-level data with cell-level mechanistic models as done in this study, insights can be gained across the scales.

Every model comes with its limitations. First, it is known that there can be time-lags between inoculation and productive infection [22]. Since these lag times vary widely among individuals, and since we wanted to avoid adding to the complexity of the model, we set the incubation period to zero. Second, since infected cells acquire a selective growth advantage only at later, symptomatic stages of the infection [31], we assumed that the presence of viral DNA did not alter the proliferation rates of infected stem cells. In addition, there is, to our knowledge, no experimental evidence regarding HPV-mediated modulation of symmetric and asymmetric division patterns in infected tissues. Third, we assumed that the interactions between virus and immune system are independent of the specific HPV strains, and that there are no synergistic or competitive effects among co-infecting types, see also [51]. Since we believe that adding these more subtle aspects would not change the main conclusion of the importance of stochasticity, we did not incorporate them into the current model. However, we plan to address these issues in future work. Fourth, a more realistic alternative to the clustered model version is provided by explicitly spatial models with lattice-based voter dynamics [52, 53]. Such a spatial model extension is subject of ongoing work. Fifth, even though the stratified squamous epithelia at different anogenital and oropharyngeal sites affected by HPV are qualitatively similar, we parametrized our model for cervical infections, and our insights regarding the role of stochastic stem cell proliferation in viral clearance may not apply to other organs. Finally, our model predicts extinction of infection with probability 1 due to the subcritical nature of the process. This is not in contradiction with the observation that a small fraction of infections persist and progress. In fact, progression from HPV infection to sustained neoplastic growth is associated with cellular changes triggered by the viral genome. These transformations are themselves stochastic processes, and hence progression only takes place in the small group of individuals where the oncogenic transformation takes place before extinction of the infected population.

## Supporting Information

S1 Text Supporting Information to Manuscript. Contains the following sections: 1. Nonparametric persistence estimators; 2. Model details; 3. MLE for branching process; 4. Identifiability issues; 5. A priori parameter estimates for cervical squamous epithelium; 6. Sensitivity of clearance time to choice of *α*; 7. Sensitivity analysis for spatial model
(PDF)
