# Supplementary material for: HPV Clearance and the Neglected Role of Stochasticity
Source: PLoS Comput Biol. 2015 Mar 13;11(3):e1004113. doi: 10.1371/journal.pcbi.1004113 (PMC4358918; doi:10.1371/journal.pcbi.1004113)
Supplement: S1 Text — Contains the following sections: 1. Nonparametric persistence estimators; 2. Model details; 3. MLE for branching process; 4. Identifiability issues; 5. A priori parameter estimates for cervical squamous epithelium; 6. Sensitivity of clearance time to choice of α; 7. Sensitivity analysis for spatial model (PDF) [file pcbi.1004113.s001.pdf]

Text S1
Supporting Information to Manuscript

HPV Clearance and the Neglected Role of Stochasticity

Marc D. Ryser[1], Evan R. Myers[2], and Rick Durrett[1]

1. Department of Mathematics, Duke University, Durham, NC, USA
2. Department of Obstetrics and Gynecology,Duke University Medical School, Durham, NC, USA

1 Nonparametric persistence estimators

First, we performed a nonparametric analysis of HPV persistence in the REACH data set. Following Moscicki and colleagues [1], we defined time to loss of infection as the time elapsed between the first positive HPV test and the first of two consecutive negative HPV tests. Thereby, a negative test was defined as loss of all HPV subtypes that had been identified during the first positive test. The corresponding Kaplan-Meier survival estimators (ignoring interval-censoring and left-truncation) are shown in Figure 1A. Comparison of these persistence curves shows that time to clearance is significantly higher in HIV-positive individuals as established by both log-rank and Wilcoxon tests ($p < 0.0005$). This is in agreement with the conclusions in [1].

However, by defining time to loss of infection as described above we lose a considerable amount of information due to interval-censoring, right-censoring and left-truncation. In fact, initiation and clearance times are only known up to intervals of length ≥ 6 months (interval-censoring), and many individuals leave the cohort study before clearing the infection (right-censoring). Furthermore, 220, or 70%, among the 313 participants have a prevalent infection (i.e. the first valid HPV test is positive), which means their time of infection initiation is unknown (left-truncation). When restricting the analysis to the 93 subjects (52 HIV-positive, 41 HIV-negative) with incident infections (i.e. the first valid HPV test is negative), we found that the two cohorts were no longer different ($p > 0.24$ by generalized log-rank test for interval-censored data). Even with the less stringent definition of clearance time as time of the first negative test result, the persistence curves of incident

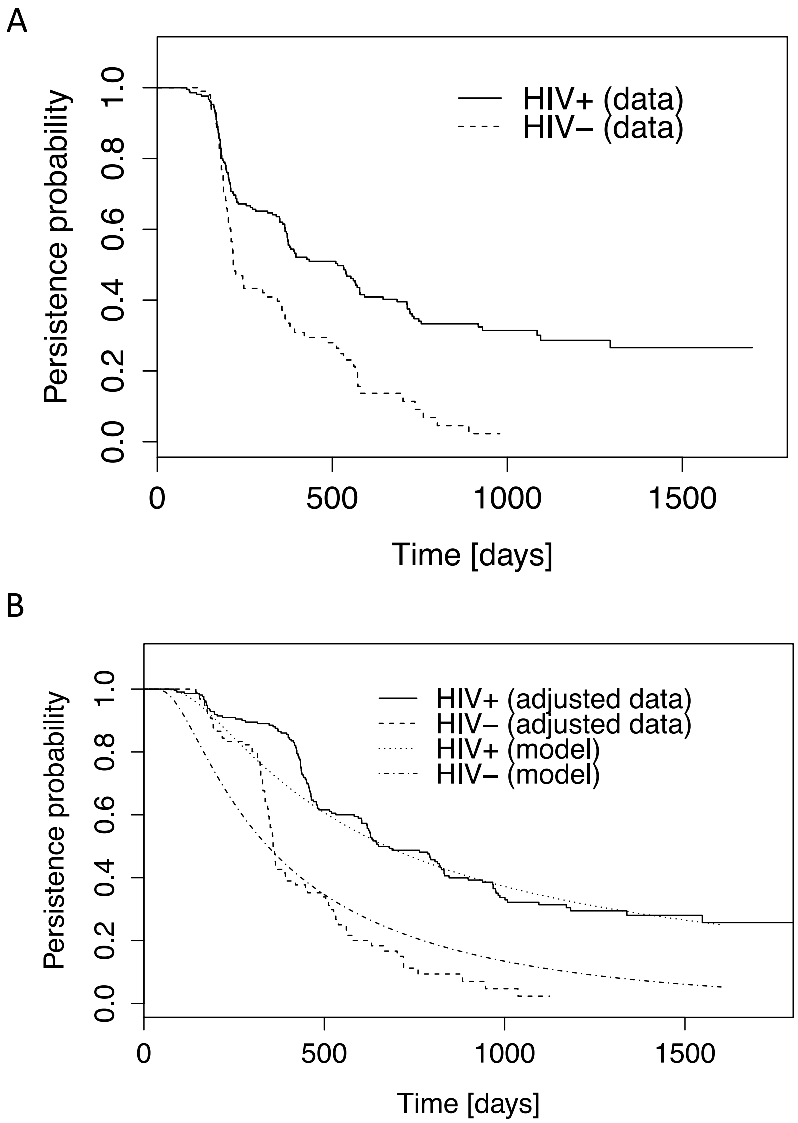

Figure 1: **Clearance time estimators. A** The nonparametric Kaplan-Meier estimators are shown for HIV-negative and HIV-positive cohorts, respectively. The Kaplan-Meier curves are obtained ignoring interval censoring and left-truncation. **B** *Adjusted data* curves: same as in panel A, but the clearance times of prevalent infections are corrected by adding model-derived estimates (median) of the times between HPV infection start and study entry. Note that interval censoring remains unaccounted for in the nonparametric estimators. *Model* curves: the parametric persistence curves are shown for both cohorts.

infections were still not significantly different ($p > 0.061$ for log-rank and Wilcoxon tests).

In summary, when restricting ourselves to the subset of incident infections, there is no significant difference between the HIV-positive and HIV-negative cohorts. However, in this analysis we neglected a lot of available information by discarding the 220 prevalent infections (70% of all cases). To incorporate prevalent infections, we need to account for the unknown time between infection start and date of first HPV test. This requires a parametric approach, as developed in the main text.

2 Model details

2.1 Replacement dynamics

If a D^* cell is eliminated, the proliferation event of the triggered stem cell is asymmetric or symmetric, as for spontaneous division. Keeping track of all possible outcomes, we have the following changes in cell balances due to D^* cell elimination:

$$D^* \to \emptyset \quad \Longleftrightarrow \begin{cases} D^* \to D & \text{at rate } \mu p_S(1-2r) \\ D^* + S \to 2D & \text{at rate } \mu p_S r \\ D^* \to S & \text{at rate } \mu p_S r \\ D^* \to D^* & \text{at rate } \mu p_{S^*}(1-2r) \\ D^* \to S^* & \text{at rate } \mu p_{S^*} r \\ S^* \to D^* & \text{at rate } \mu p_{S^*} r. \end{cases} \tag{1}$$

While S and S^* cell proliferation caused by D^* cell elimination is identical to spontaneous division, S^* cell elimination is assumed to trigger a symmetric division resulting in two identical S or S^* copies, respectively. In fact, this assumption is necessary to ensure conservation of the total number of stem cells in the homeostatic regime: if an S^* cell division were to trigger all three possible division patterns, the total number of stem cells in the tissue would not be conserved over time. Keeping track of all possible outcomes after S^* cell elimination, we find the following rates

$$S^* \to \emptyset \quad \Longleftrightarrow \begin{cases} S^* \to S & \text{at rate } \mu\, p_S \\ S^* \to S^* & \text{at rate } \mu\, p_{S^*}. \end{cases} \tag{2}$$

2.2 Spatial Model

The stochastic process corresponding to the spatial model dynamics in the S^*-D^* plane is summarized in Figure 2. In Figure 3, three different realizations of the process are shown for the representative parameter set $\alpha = 0.12$ (chosen) and $\mu = 1.3 \cdot 10^{-3}$, and $n_0 = 37$ (both maximum likelihood estimates from the branching process model). See caption for remaining parameters.

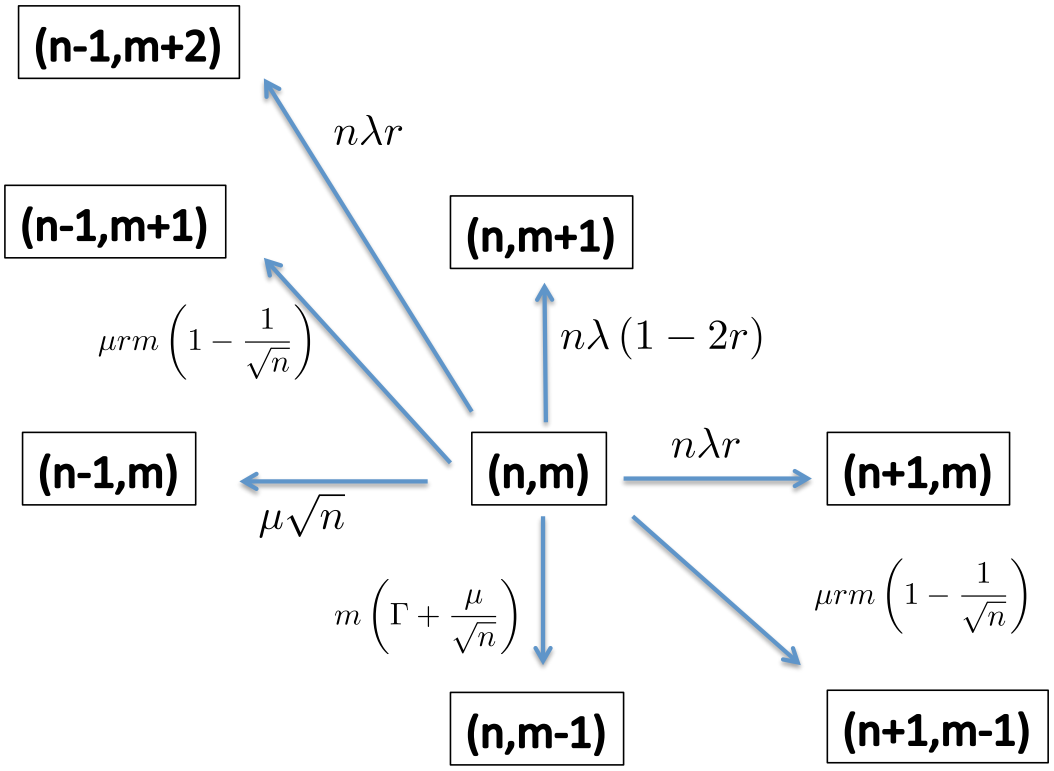

Figure 2: **Stochastic dynamics for the S^* (n) and D^* (m) populations.** The stochastic jump rates are derived from the combination of homeostatic division events, cell elimination by the immune system, and transition into the suprabasal compartment by differentiated daughter cells.

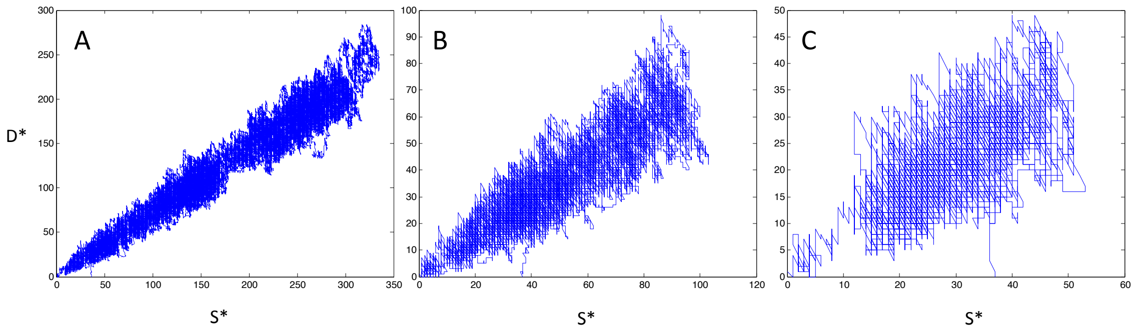

Figure 3: **Realizations of the clustering process.** Three different realizations of the dynamics depicted in Figure 2 are shown. The initial conditions and parameters are as follows: $S^*(t=0) = 37$, $D^*(t=0) = 0$, $\alpha = 0.12$, $\mu = 1.3 \cdot 10^{-3}$, $r = 0.18$, $\rho = 0.58$.

3 MLE for Branching Process

In this section, we provide the details for the parameter inference through MLE. To derive the likelihood function for the extinction time of the subcritical branching process

$$S^* \to \begin{cases} S^* + S^* & \text{at rate } \lambda r, \\ \emptyset & \text{at rate } \lambda r + \mu, \end{cases} \tag{3}$$

we need to derive the exact distributions. We denote by τ_{n_0} the time to extinction of the infection process with initial condition $n_{s^*}(t=0) = n_0$. We introduce now the survival function

$$\mathcal{F}(t) = \mathbb{P}\left(\tau_{n_0} > t\right) = \mathbb{P}\left(n_{s^*}(t) \geq 0 | n_{s^*}(0) = n_0\right).$$

Since the n_0 families are independent, we have

$$\mathcal{F}(t) = 1 - \left[\mathbb{P}\left(\tau_1 \leq t\right)\right]^{n_0} = 1 - \left(1 - \mathcal{F}_1(t)\right)^{n_0}, \tag{4}$$

where the one-particle survival function $\mathcal{F}_1$ is

$$\mathcal{F}_1(t) = \begin{cases} \left(1 + \frac{\lambda r + \mu}{\mu}\left(e^{\mu t} - 1\right)\right)^{-1}, & \mu > 0, \\ \left(1 + \lambda r t\right)^{-1}, & \mu = 0. \end{cases} \tag{5}$$

Introducing the notation $A \equiv (\lambda r + \mu)/\mu$ for $\mu > 0$, we can write the survival function as

$$\mathcal{F}(t) = \begin{cases} 1 - \left(\frac{\lambda r t}{1 + \lambda r t}\right)^{n_0}, & \mu = 0, \\ 1 - \left(\frac{A\left(e^{\mu t} - 1\right)}{1 + A\left(e^{\mu t} - 1\right)}\right)^{n_0}, & \mu > 0. \end{cases} \tag{6}$$

Differentiating yields the probability density functions

$$f(t) = \begin{cases} \frac{n_0 \lambda^{n_0} r^{n_0} t^{n_0 - 1}}{(1 + \lambda r t)^{n_0 + 1}}, & \mu = 0, \\ \frac{n_0 A^{n_0} \mu e^{\mu t}\left(e^{\mu t} - 1\right)^{n_0 - 1}}{\left(1 + A\left(e^{\mu t} - 1\right)\right)^{n_0 + 1}}, & \mu > 0. \end{cases} \tag{7}$$

We can now combine (6) and (7) with the data from the REACH study to derive the likelihood function. But first, we need to understand the data format. The data is given in the form $\omega = (p_j, d_j, t_j^i, t_j^f, \tilde{t}_j^i, \tilde{t}_j^f)$, for $j = 1, \ldots, n$, where

- $p_j = 1$ if the infection was present at the first HPV test during the study; $p_j = 0$ if the infection was not yet present at the first HPV test.

- $d_j = 1$ if the infection is cleared during the study; $d_j = 0$ if the subject is lost to follow-up before clearing the infection

- t^i_j is time of the first positive HPV test.

- t^f_j: if $d_j = 1$, then it is the time of the first of two negative results (if $d_j = 1$); if $d_j = 0$, then it is the censoring time.

- We denote by $\tilde{t}^{i/f}_j$ the time of the visit preceding the visit at $t^{i/f}_j$.

To make the notation lighter, we omit the subscript j in the following calculations. We introduce the notation $\Delta t^i \equiv t^i - \tilde{t}^i$ and $\Delta t^f \equiv t^f - \tilde{t}^f$. See also Figure 4 for a visualization of the time sequence.

We introduce the random variable τ which is the time from infection start to clearing of the virus.

Denote by f and F the pdf and cdf of the time to clearance τ, and define the survivor function $\mathcal{F} \equiv 1 - F$. Furthermore, define g and G to be the pdf and cdf of the censoring times (counted since start of the study), and define the corresponding survivor funcion $\mathcal{G} \equiv 1 - G$. Finally, denote by t_0 the study begin - in fact, the censoring events are counted from study begin onward.

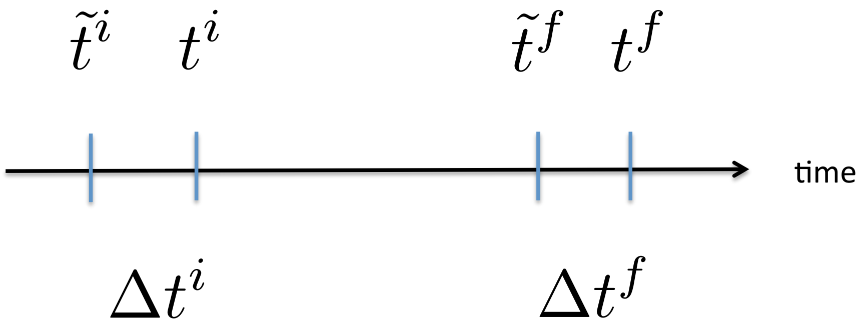

Figure 4: **Events**

We discuss now the different contributions to the likelihood function.

Prevalent infections ($p = 1$)**.** If the infection was present at the first HPV test (prevalent infection), then the infection started σ days before study begin, i.e. only $\tau - \sigma$ days can

be observed until clearance (recall that τ is the time to clearance counted from onset of the infection, not the start of the study). Therefore, the contributions to the likelihood function are

$$\mathbb{P}\left(\tau - \sigma > t^f - t^i\right) g(t^f - t_0), \quad d = 0, \tag{8}$$

and

$$\mathbb{P}\left(\tau - \sigma \in [\tilde{t}^f - t^i, t^f - t^i]\right) \mathcal{G}(t^f - t_0), \quad d = 1. \tag{9}$$

It remains to get a handle on the distribution of $\tau - \sigma$. In fact, once we know the length of the infection period, i.e. we condition on $\{\tau = t\}$, then its hidden length is uniformly distributed over t because we assume constant arrival rate of new infections,

$$\mathbb{P}\left(\sigma = s | \tau = t\right) = \frac{1}{t} \mathbb{1}_{\{0 \le s \le t\}}(s),$$

and

$$\mathbb{P}\left(\sigma < s | \tau = t\right) = \left(\frac{s}{t} \wedge 1\right) \mathbb{1}_{\{0 \le s\}}(s).$$

With this, we derive (note that $\in ds$ is short for $\in [s, s + ds)$)

$$\begin{aligned}
\mathbb{P}\left(\tau - \sigma \in ds\right) &= \int_0^\infty \mathbb{P}\left(\tau - \sigma \in ds | \tau \in dt\right) \mathbb{P}\left(\tau \in dt\right) \\
&= \int_0^\infty \frac{1}{t} \mathbb{1}_{\{0 \le s \le t\}}(s) f(t) dt \\
&= \int_s^\infty \frac{1}{t} f(t) dt,
\end{aligned}$$

and, similarly,

$$\mathbb{P}\left(\tau - \sigma > s\right) = \int_s^\infty \left(1 - \frac{s}{t}\right) f(t) dt.$$

To make sure our calculations are correct, we check, using a change of variables, that the pdf integrates to 1,

$$\int_0^\infty \int_s^\infty \frac{f(t)}{t} dt ds = \int_0^\infty \int_0^t \frac{f(t)}{t} ds dt = 1.$$

Incident infections ($p = 0$). In this case, the first HPV test of the study is negative, i.e. the infection starts after the first visit (an incident infection). More precisely, it started $\hat{\sigma}$ days before the first positive test at time t^i, but unlike σ which is supported on $[0, \infty)$, the time $\hat{\sigma}$ cannot exceed Δt^i (because if it did exceed Δt^i, then the visit at $\tilde{t}^i$ would have been positive). The contributions to the likelihood function are now

$$\mathbb{P}\left(\tau - \hat{\sigma} > t^f - t^i\right) g(t^f - t_0), \quad d = 0, \tag{10}$$

and

$$\mathbb{P}\left(\tau - \hat{\sigma} \in [\tilde{t}^f - t^i, t^f - \tilde{t}^i]\right) \mathcal{G}(t^f - t_0), \quad d = 1. \tag{11}$$

It remains to compute the distribution of $\hat{\sigma}$:
First, we note that if $t > \Delta t^i$, then

$$\mathbb{P}(\hat{\sigma} = s | \tau = t) = \begin{cases} \left(\Delta t^i\right)^{-1} & \text{if } 0 \leq s \leq \Delta t^i \\ 0 & \text{otherwise} \end{cases}$$

and if $t \leq \Delta t^i$, then

$$\mathbb{P}(\hat{\sigma} = s | \tau = t) = \begin{cases} t^{-1} & \text{if } 0 \leq s \leq t \\ 0 & \text{otherwise .} \end{cases}$$

Combining the two, we find now (note that $a \wedge b$ is short for $\min\{a, b\}$)

$$\mathbb{P}(\hat{\sigma} = s | \tau = t) = \frac{1}{t \wedge \Delta t^i} \mathbb{1}_{\{0 \leq s \leq t \wedge \Delta t^i\}}(s), \tag{12}$$

and, similarly,

$$\mathbb{P}\left(\hat{\sigma} < s | \tau = t\right) = \left(1 \wedge \frac{s}{t \wedge \Delta t^i}\right) \mathbb{1}_{\{s \geq 0\}}(s).$$

Using these formulae, we can now compute

$$\begin{aligned} \mathbb{P}\left(\tau - \hat{\sigma} = z\right) &= \int_0^\infty \mathbb{P}\left(\tau - \hat{\sigma} = z | \tau = t\right) \mathbb{P}\left(\tau \in dt\right) \\ &= \int_0^\infty \mathbb{P}\left(\hat{\sigma} = t - z | \tau = t\right) f(t) dt \\ &= \int_0^\infty \frac{1}{t \wedge \Delta t^i} \mathbb{1}_{\{0 \leq t - z \leq t \wedge \Delta t^i\}}(z) f(t) dt \\ &= \int_0^\infty \frac{1}{t \wedge \Delta t^i} \mathbb{1}_{\{z \leq t \leq z + t \wedge \Delta t^i\}}(z) f(t) dt \\ &= \int_z^\infty \frac{1}{t \wedge \Delta t^i} \mathbb{1}_{\{t \leq z + t \wedge \Delta t^i\}}(z) f(t) dt, \end{aligned} \tag{13}$$

and

$$\mathbb{P}\left(\tau - \hat{\sigma} > z\right) = \int_z^\infty \left(1 \wedge \frac{t - z}{\Delta t^i \wedge t}\right) f(t) dt.$$

Here too, the sanity check pans out, and the pdf integrates to 1. The calculation is messy, and we omit it here.

Finally, we can assemble the complete log- likelihood function. To this end, we reintroduce the subscripts in the time variables, and combine (8), (9), (13) and (11) to obtain

$$\begin{aligned}\mathcal{L}(\theta) = &\sum_{\{j\,:\,p_j=1,d_j=0\}} \log\ \mathbb{P}_\theta\left(\tau-\sigma > t_j^f - t_j^i\right) \\ &+ \sum_{\{j\,:\,p_j=d_j=1\}} \log\ \mathbb{P}_\theta\left(\tau-\sigma \in [\tilde{t}_j^f - t_j^i, t_j^f - t_j^i]\right) \\ &+ \sum_{\{j\,:\,p_j=d_j=0\}} \log\ \mathbb{P}_\theta\left(\tau-\hat{\sigma}_j > t_j^f - t_j^i\right) \\ &+ \sum_{\{j\,:\,p_j=0,d_j=1\}} \log\ \mathbb{P}_\theta\left(\tau-\hat{\sigma}_j \in [\tilde{t}_j^f - t_j^i, t_j^f - \tilde{t}_j^i]\right).\end{aligned} \tag{14}$$

In the above expression, $\theta = (\mu, \lambda, r, n_0)$ are the model parameters, $\mathbb{P}_\theta$ is the probability assuming parameter set θ, and we have added a subscript to $\hat{\sigma}_j$ to emphasize its dependence on Δt_j^i, see (22).

Finally, to infer the most likely parameter set, we maximized the likelihood function (14) using the statistical software R [2]. We did this by combining a coarse grid search across prior parameter ranges (see Section 5) with the built-in local optimization routine *optim()*.

4 Identifiability Issues

Even though there are no apparent identifiability issues in the probability density function (7), when performing the MLE for the branching process model, we found that equally good fits were obtained for a fixed ratio α/n, where $\alpha = \lambda r$ and n is the size of the initial infection. In other words, the distribution depends primarily on α/n, and is insensitive to the absolute values of α and n. To provide a theoretical explanation for this finding, we start by performing a series expansion in μ of the density f in (7)

$$f(t,\alpha,n,\mu) = \alpha n \left(\frac{\alpha t}{1+\alpha t}\right)^{n-1} \frac{1}{(1+\alpha t)^2} + \mathcal{O}(\mu). \tag{15}$$

Next, we zoom out by rescaling time as $\hat{t} = t\alpha/n$, and obtain the rescaled density $\hat{f}$

$$\hat{f}(\hat{t},\alpha,n,\mu) = n^2 \left(\frac{\hat{t}n}{1+\hat{t}n}\right)^{n-1} \frac{1}{\left(1+\hat{t}n\right)^2} + \mathcal{O}(\mu). \tag{16}$$

Importantly, the lowest order term converges to

$$\frac{e^{-1/\hat{t}}}{\hat{t}^2}, \quad n \to \infty, \tag{17}$$

and numerical experimentation shows that convergence is rapid, with good results achieved for $n = \mathcal{O}(10)$. Next, we replace $\alpha \to \beta$ and $n \to n\frac{\beta}{\alpha}$ to conserve the ratio α/n and calculate

$$\hat{f}(\hat{t}, \beta, n\frac{\beta}{\alpha}, \mu) = (n\frac{\beta}{\alpha})^2 \left(\frac{\hat{t}n\frac{\beta}{\alpha}}{1 + \hat{t}n\frac{\beta}{\alpha}} \right)^{n\frac{\beta}{\alpha}-1} \frac{1}{\left(1 + \hat{t}n\frac{\beta}{\alpha}\right)^2} + \mathcal{O}(\mu). \tag{18}$$

Again, the lowest order term converges to

$$\frac{e^{-\hat{t}}}{\hat{t}^2}, \quad n \to \infty, \tag{19}$$

and we conclude that to first order in μ (and for t and n large enough)

$$f(t, \alpha, n, \mu) \approx f(t, \beta, n\frac{\beta}{\alpha}, \mu). \tag{20}$$

Since the longitudinal study used for parameter inference contains mostly large time points (order of months), and the inferred μ is small, this provides insight as to why the inference is insensitive to the absolute values of α and n, and primarily depends on their ratio.

5 A Priori Parameter Estimates for cervical squamous epithelium

For the parameter estimation by MLE as well as the sensitivity analysis in Section 7 we use a priori estimates for the following model parameters:

- ρ: the fraction of proliferative cells in the basal layer,
- Γ: the exit rate of D and D^* cells from the basal layer,
- λ: the proliferation rate of stem cells,
- r: the probability of symmetric division.

These parameters are not completely independent because there are two constraints imposed by the homeostatic equilibrium of the epithelium. First, the basal layer is in a stationary state: arrival of new D cells produced by S cells is balanced by the exit of D cells into the parabasal layers, i.e.

$$\Gamma(1-\rho) = \rho\lambda. \tag{21}$$

Furthermore, we know that there is periodic renewal of the entire epithelium, with a renewal time of ΔT, and hence the number of upwards migrating cells over ΔT has to satisfy

$$(1-\rho)\Gamma\Delta T = H, \tag{22}$$

where H is the height (in numbers of cells) of the stratified squamous epithelium. Combining (21) and (22) we find

$$\lambda = \frac{H}{\rho \Delta T}. \tag{23}$$

To derive the various a priori estimates, we use (21) and (23) together with literature estimates as follows.

- The thickness H of the squamous epithelium is estimated using data from [3]. In this study, the authors use histological sections of excised specimen to find an average thickness of $360 \pm 100\mu m$. They then split the height into 4 equal parts, and assess the cell heights in μm (from bottom to top quarter of thickness): 15 ± 5, 17 ± 5.1, 14 ± 4.7 and 14 ± 4.8. Using these averages, we find that the epithelium stacks approximately

  $$H \sim 24 \text{ cells}$$

  high from basement membrane to lumen of the cervix.

- In [4] the authors use BrdU staining to find that the fraction of cells in S-phase is 10.7% in the lower third ($\sim$ 8 cells) of the epithelium. Since all proliferative cells in the healthy tissue are located in the basal layer, this means that about 75% of basal cells are mitotically active, i.e. $\rho = 75\%$. Of note, estimates for the mouse tail epidermis [5] and mouse esophageal epithelium [6] are $\rho = 22\%$ and $\rho = 65\%$, respectively. Due to this wide spread in murine data, and the lack of reliable experimental estimates in humans, we assume the following a priori bounds:

  $$\rho \in [0.2, 0.8].$$

- Literature estimates for the time of complete renewal of the squamous epithelium of the cervix vary between 3 and 6 weeks [7,8],

  $$\Delta T \in [21, 42] \text{ days}.$$

- Using the above estimates, we find now an a priori estimate for $\lambda \in [0.29, 9.5]$/day. The upper bound is clearly too big and we assume that the minimal time for a complete cell cycle is 1 day, leading to the following prior range,

  $$\lambda \in [0.29, 1] \text{ day}^1.$$

- Estimates for r, the probability of a stem cell dividing into two stem cells (or into two differentiated cells), are not available for human tissues. In the mouse tail epidermis [5] and the mouse esophagus this probability was estimated to be $r = 0.1$ and $r = 0.09$, respectively. We assume a priori bounds of

  $$r \in [0.05, 0.25].$$

- Finally, combining the a priori estimates for λ and r, we find the prior range for α used in this study,

$$\alpha \in [0.015, 0.25] \text{ day}^{-1}.$$

6 Sensitivity of Clerance Time to Choice of α

In Figure 3 of the main text, we showed the clearance time distributions for an intermediate value of $\alpha = 0.14$. In Figure 5, we show the clearance time distributions over the entire prior range of $\alpha \in [0.01, 0.25]$, both for HPV-negative individuals (panel A) and HPV-positive individuals (panel B). In particular, we observe that the respective distributions are very similar across the entire range.

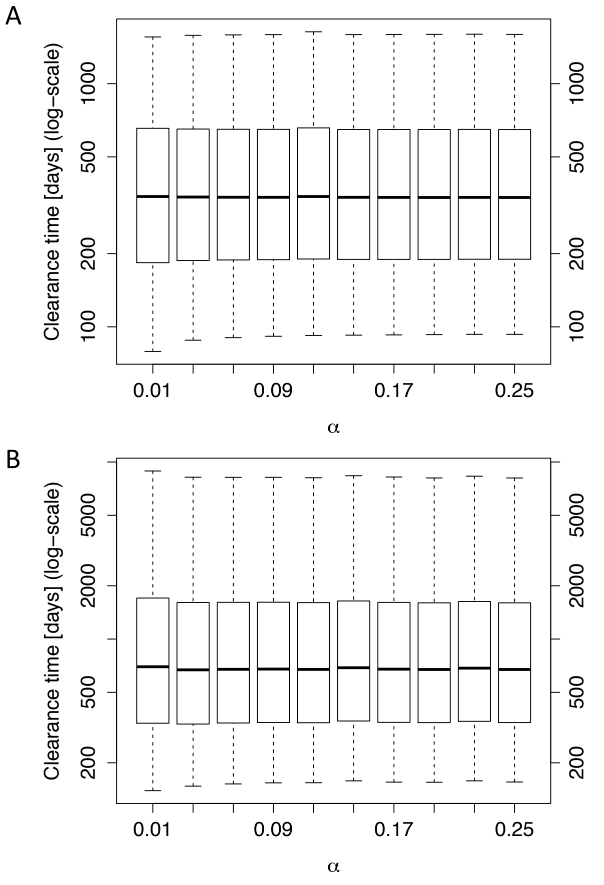

Figure 5: **Sensitivity of clearance time distribution to choice of α.** The clearance time distributions for the non-spatial branching process model in HIV-negative individuals (A), and HPV-positive individuals (B), are shown for 10 different values of α spanning the prior range $[0.01, 0.25]$.

7 Sensitivity Analysis for Spatial Model

In Figure 5 of the main text we assessed the sensitivity of the spatial clustering model to the immune capacity μ. Here, we do a more extensive sensitivity analysis by performing 5000 simulations, and for each run the parameters are drawn at random from the following a priori intervals: $r \in [0.05, 0.15]$, $\alpha = r\lambda \in [0.015, 0.25]$, $\rho \in [0.2, 0.8]$, $\mu \in [0.001, 0.0018]$, the latter being an uncertainty interval around $\hat{\mu}_{-}$, the estimated immune capacity of the HIV-negative cohort. Furthermore, we only allowed for parameter sets that are compatible with the a priori intervals for H and ΔT, see Section 5. The initial infection size, n_0 was obtained by calculating n_0 as a function of α as determined in the branching process model, see Figure 3A in the main text. In Figure 6, the clearance time distributions are compared for the non-spatial branching process model (A), the spatially clustered model with fixed parameters (B) and the spatially clustered model with random parameters (C). In particular, by comparing (B) and (C) we conclude that drawing the parameters at random within their prior ranges does not affect the clearance time distribution substantially.

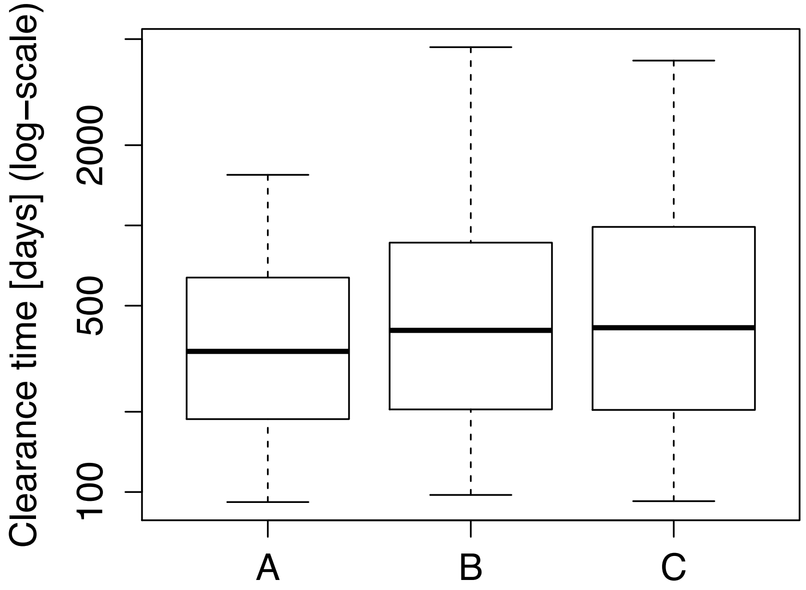

Figure 6: **Sensitivity analysis.** The clearance time distributions for the non-spatial branching process model (A), the stochastic model with fixed parameter values (B), and the stochastic model with random parameter values (C) are shown. The latter was obtained by running 5000 simulations with random parameter values, see text for details on parameter ranges.

References

[1] Moscicki A, Ellenberg J, Farhat S, Xu J (2004) Persistence of human papillomavirus infection in HIV-infected and-uninfected adolescent girls: risk factors and differences, by phylogenetic type. J Infect Dis 190: 37–45.

[2] R Core Team (2013) R: A Language and Environment for Statistical Computing. R Foundation for Statistical Computing, Vienna, Austria. URL http://www.R-project.org/.

[3] Walker D, Brown B, Blackett A, Tidy J, Smallwood R (2003) A study of the morphological parameters of cervical squamous epithelium. Physiol Meas 24: 121.

[4] Fukuda K, Iwasaka T, Hachisuga T, Sugimori H, Tsugitomi H, et al. (1990) Immunocytochemical detection of S-phase cells in normal and neoplastic cervical epithelium by anti-BrdU monoclonal antibody. Anal Quant Cytol Histol 12: 135–138.

[5] Clayton E, Doupé D, Klein A, Winton D, Simons B, et al. (2007) A single type of progenitor cell maintains normal epidermis. Nature 446: 185–189.

[6] Doupé DP, Alcolea MP, Roshan A, Zhang G, Klein AM, et al. (2012) A single progenitor population switches behavior to maintain and repair esophageal epithelium. Science 337: 1091–1093.

[7] Stanley M (2006) Immune responses to human papillomavirus. Vaccine 24: S16–S22.

[8] Stanley M (2010) Pathology and epidemiology of HPV infection in females. Gynecol Oncol 117: S5–S10.
